# Is high tibial osteotomy better than proximal fibula osteotomy for treating knee osteoarthritis? A protocol for a systematic review and meta-analysis of clinical controlled trials

**DOI:** 10.1097/MD.0000000000018910

**Published:** 2020-01-24

**Authors:** Hetao Huang, Sicong Huang, Guihong Liang, Lingfeng Zeng, Jianke Pan, Weiyi Yang, Hongyun Chen, Jun Liu, Biqi Pan

**Affiliations:** a Second School of Clinical Medicine, Guangzhou University of Chinese Medicine; b Department of Orthopaedics, Foshan Second People's Hospital; c Department of Orthopaedics, Second Affiliated Hospital of Guangzhou University of Chinese Medicine, Guangdong Provincial Hospital of Chinese Medicine; d Department of Traditional Chinese Medicine, GuangDong Women and Children Hospital, China.

**Keywords:** HTO, KOA, meta-analysis, PFO

## Abstract

**Background:**

Knee osteoarthritis (KOA) is a common disease in the elderly, which seriously reduces the quality of life of patients and increases the social burden. proximal fibula osteotomy (PFO) and high tibial osteotomy (HTO) are effective methods to treat KOA. However, it is not entirely clear which method has the advantage. Therefore, we evaluated the efficacy and safety of HTO and PFO in the treatment of KOA.

**Methods:**

Randomized controlled trials from online databases including PubMed, Embase, the Cochrane Library, China National Knowledge Infrastructure, Chinese Scientific Journal Database, Wanfang Data and Chinese Biomedical Literature Database that compared the efficacy of HTO and PFO in the treatment of KOA were retrieved. The main outcomes included hospital for special surgery (HSS) knee scores, knee society knee scoring system (KSS) score, visual analog scale (VAS) knee pain scores, western ontario and McMaster universities osteoarthritis index score, operation time, intraoperative bleeding volume, hospitalization time, complications. The Cochrane risk of bias tool was used to assess methodological quality.

**Results:**

The literature will provide a high-quality analysis of the current evidence supporting HTO for KOA based on various comprehensive assessments including HSS scores, KSS score, VAS scores, western Ontario and McMaster universities osteoarthritis index score, operation time, intraoperative bleeding volume, hospitalization time, and complications.

**Conclusion:**

This proposed systematic review will provide up-to-date evidence to assess the effect of HTO in the treatment for patients with KOA.

## 1 Introduction

Knee osteoarthritis (KOA) is a common disease in orthopedic clinics and occurs mostly in middle-aged and elderly people. It is 1 of the most common causes of joint pain, functional loss and disability in adults.^[1–2]^ The main clinical manifestations are joint pain, joint deformity, and limited movement. Pathological changes are mainly articular cartilage injury, osteophyte formation, degeneration, and injury of the subchondral bone and meniscus.^[3]^ KOA is a chronic, progressive disease that seriously reduces the quality of life of patients and places a huge economic burden on their families and society.^[4]^ Authoritative epidemiological studies have shown that 18% of women and 10% of men over 60 years of age are affected by osteoarthritis worldwide, and KOA accounts for the majority.^[5]^ At present, the specific pathogenesis of KOA is still unclear, and treatment methods are diverse. Western medicine mainly treats the disease with nonsteroidal antiinflammatory drugs and arthroscopic debridement, high tibial osteotomy (HTO), proximal fibular osteotomy (PFO), unicondylar knee arthroplasty, and total knee arthroplasty. Surgical treatment is the main treatment known to have a definitive clinical effect, but there are still some disputes on the timing of the application of various treatment methods, and there are some differences in surgical indications.^[6–10]^

In recent years, with the popularization of the concept of KOA ladder therapy, the number of operations for HTO and PFO has increased year by year. Some studies^[11]^ believe that PFO has the advantages of early surgical effect, less trauma and shorter operation time, but the long-term effect of HTO is better than that of PFO. Due to the lack of sufficient clinical evidence, it is impossible to determine the advantages and disadvantages of HTO and PFO. Therefore, the author uses the Cochrane system evaluation method to collect high-quality research literature in authoritative databases at home and abroad and to systematically evaluate the efficacy and safety of HTO and PFO in the treatment of KOA with meta-analysis to achieve a better result. The current study was conducted to provide a reference for clinical decision-making.

## 2 Methods

This systematic review protocol has been registered on PROSPERO (https://www.crd.york.ac.uk/prospero/display_record.php?RecordID=146426). The registration number is CRD42019146426. This protocol was performed in accordance with the preferred reporting items for systematic reviews and metaanalysis protocol. Ethical approval is unnecessary because this is a literature-based study.

### 2.1 Data sources and search strategy

Seven databases, including the Cochrane library, PubMed, EMBASE, Chinese Biomedical Literature Database, Chinese Journal Full-Text Database, Wanfang Resources Database, Weipu Journal Database, were investigated from inception to June 1, 2019. The reference list of retrieved papers was also reviewed. The following search terms were used individually or in combination: ‘HTO’, ‘high tibial osteotomy’, ‘PFO’, ‘proximal fibular osteotomy’, and ‘KOA’. To increase the search range, no date or language limits were imposed. Additionally, no restrictions on population characteristics were imposed.

### 2.2 Inclusion criteria and study selection

#### 2.2.1 Participants

Only published articles enrolling adult participants with a diagnosis of KOA will be included. The patient's gender, age, and grades of KOA will not be limited.

#### 2.2.2 Interventions

The intervention group will have treated with a HTO.

#### 2.2.3 Comparisons

The control group will have received PFO.

#### 2.2.4 Outcomes

The primary outcomes of this meta-analysis were “ hospital for special surgery scores”, and the secondary outcomes were “ knee society knee scoring system score”, “ visual analog scale scores”, “ western Ontario and McMaster universities osteoarthritis index score”, “operation time”, “intraoperative bleeding volume”, “hospitalization time”, and “complications.”

#### 2.2.5 Study design

**Figure 1:**
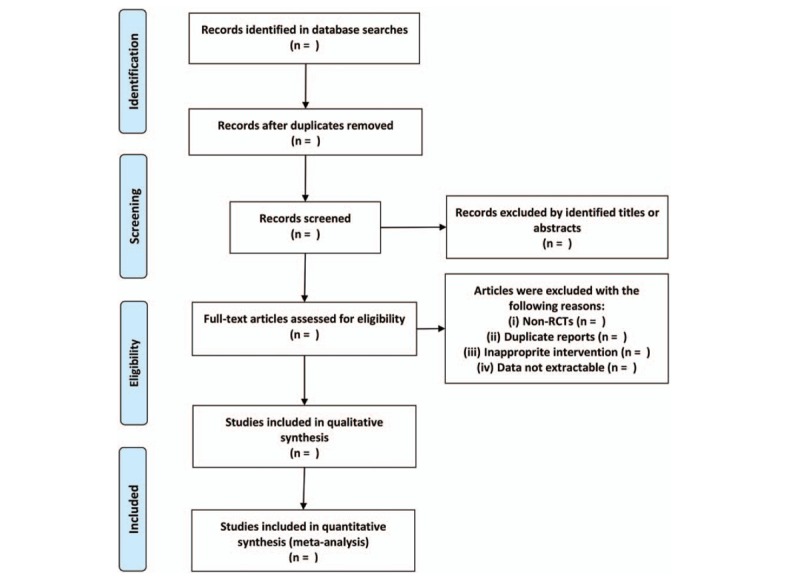
Flow diagram of study selection.

Randomized controlled trials (RCTs) will be considered eligible for our study. Articles will be excluded if they are case reports, letters, editorials, and nonhuman studies. The flow diagram of the study selection is shown in Figure 1.

### 2.3 Data extraction

Data extraction included the first author's name, year of publication, sample size, diagnostic criteria, age, and sex of the participants, details of the intervention and control conditions, treatment duration and outcome measurements for each study. Two authors (HTH, SCH) independently conducted the data extraction according to predefined criteria. Any uncertainty was resolved through discussion with another author (BQP). The reasons for exclusion were recorded. The data were extracted from the included RCTs to a predefined Excel table (Microsoft Corp, Redmond, WA) and cross-checked by the 2 reviewers (HTH, GHL). In the event of missing data, we will attempt to contact the corresponding authors for details.

### 2.4 Assessment of methodologic quality

Two authors (HTH, LFZ) independently assessed the methodological quality of each trial according to the standards advised by the Cochrane Handbook.^[12]^ Disagreements, if any, were resolved by discussion and reached consensus through a third reviewer (BQP). The risk of bias was evaluated for each study by assessing the randomization process, the treatment allocation concealment, blinding of participants and personnel, blinding of outcome assessment, the completeness of the data, the reporting of results and other biases. Selective reporting bias was judged according to the published protocols for the registered clinical trials that were contained on the Chinese clinical trial registry (http://www.chictr.org) and international clinical trial registry of the US National Institutes of Health (http://clinicaltrials.gov) websites. We compared the outcome measures between the study protocol and the final published trial.

### 2.5 Data analysis

Data analysis was carried out using Review Manager software (V.5.3) provided by the Cochrane Collaboration. Given the characteristics of the extracted data in the review, continuous outcomes were expressed as the mean differences with 95% confidence intervals (CIs). Differences in categorical variables were expressed as risk ratio values and 95% CIs. Heterogeneity was assessed by means of *I*^*2*^ statistics. *I*^*2*^≥50% represented high heterogeneity. A standardized mean difference was used when the studies included in the meta-analysis assessed the outcome based on different scales (eg, visual analog scale [VAS] 0–10 and VAS 0–100). Initially, a fixed-effect model would be used to compare the outcomes, unless the heterogeneity tests indicated that the *I*^2^ statistic ≥50% and substantial heterogeneity existed between studies; in this case, the reasons for this heterogeneity would be searched for and a random-effect model would be used for comparison. The subgroup analysis was undertaken according to prespecified criteria to investigate heterogeneous results or to determine the effect of prespecified criteria on the pooled estimate. Publication bias was analyzed by funnel plot analysis if sufficient studies (n≥10) were found.

### 2.6 GRADE the evidence

The grading recommendations assessment, development and evaluation (GRADE) system was used to evaluate the quality of the evidence for each outcome. GRADE-pro GDT Online Tools (available on https://gradepro.org/) were used to evaluate the evidence regarding the included outcomes. Initially, RCTs were considered to be of high confidence in estimating an effect, and observational studies were considered to be of low confidence in estimating an effect. The reasons that may decrease the level of confidence included risk of bias, inconsistency, indirectness, imprecision, and publication bias. The reasons that may increase the level of confidence included a large effect, dose response, and accounting for all plausible residual confounding and bias. The GRADE evidence was divided into the following categories:

(1) High-quality evidence, which indicated that further research was unlikely to change the confidence in the estimate of the effect;
(2) Moderate-quality evidence, which indicated that further research was likely to have an important impact on the confidence in the estimate of the effect and may change the estimate;
(3) Low-quality evidence, which indicated that further research was likely to have an important impact on confidence in the estimate of the effect and was likely to change the estimate; and
(4) Very low-quality evidence, which indicated that we were very uncertain about the results.

## 3 Discussion

HTO has been promoted by Coventry,^[13]^ and it has been developed for more than 50 years. It mainly transfers the lower limb force line axis to the lateral compartment, thereby reducing the load of the medial compartment and delaying the progress of KOA.^[14]^ A study has shown that regeneration can also occur in the articular cavity after lower limb force line correction, including articular cartilage regeneration.^[15]^ The indications^[12,16]^ for HTO include the following: ① X-ray findings of full-length weight-bearing position of lower extremities: varus deformity of the knee joint >5 degrees, accompanied by osteoarthritis of knee joint; ② osteoarthritis in the medial compartment, the lateral compartment is intact, and conservative treatment was ineffective for more than 6 months; ③ varus angle of knee joint <20 degrees, joint mobility >9; and ④ the patient can tolerate operation and exercise the affected limb function 4 to 6 weeks after operation (in the case of nonload).

PFO was first proposed by Starr in 1945.^[17]^ Several clinical studies have found that knee pain symptoms in KOA patients can be significantly alleviated or may even disappear after PFO.^[18–20]^ The indications for PFO include the following:

(1) The main clinical manifestations were inflammation of the medial compartment of the knee joint, active pain or resting pain of the knee joint, tenderness of the medial or anteromedial fingers of the knee joint;
(2) X-ray examination showed that the medial space of the knee joint became narrower, force lines of the lower limbs were measured on the X-ray film of the weight-bearing position, and varus deformity of the knee existed. These patients can be treated with PFO.

As the systematic review is based on the secondary research of published literature, there are undeniable methodological defects. In addition, the quality of the included studies determines the quality level and reliability of the final results. We will begin to conduct the review when the necessary trials are met, and all operating procedures will be performed in accordance of Cochrane Handbook to ensure that the provided information is helpful for clinicians and patients.

## Acknowledgments

We would like to thank Professor Holger Schulenemann, Chairman of GRADE Working Group, Department of Clinical Epidemiology and Biomedical Statistics, McMaster University, Canada; Professor Li Youping, Director of Cochrane Center in China; Professor Yang Kehu, Director of GRADE Center in China; Professor Tian Jinhui, Evidence-based Medicine Center of Lanzhou University for their training on Cochrane system evaluation and grade system knowledge.

## Author contributions

**Conceptualization**: Hetao Huang, Biqi Pan.

**Data curation**: Hetao Huang, Sicong Huang, Guihong Liang, Lingfeng Zeng.

**Formal analysis**: Hetao Huang, Sicong Huang, Lingfeng Zeng.

**Funding acquisition**: Jianke Pan, Weiyi Yang, Hongyun Chen.

**Investigation**: Hetao Huang, Jun Liu.

**Methodology**: Guihong Liang, Lingfeng Zeng, Jianke Pan.

**Project administration**: Hetao Huang, Sicong Huang, Guihong Liang, Biqi Pan.

**Resources**: Jianke Pan, Weiyi Yang, Hongyun Chen.

**Software**: Hetao Huang, Sicong Huang, Jianke Pan.

**Supervision**: Jianke Pan, Weiyi Yang.

**Validation**: Weiyi Yang, Biqi Pan.

**Visualization:** Jianke Pan.

**Writing – original draft**: Hetao Huang, Sicong Huang.

**Writing – review & editing:** Biqi Pan.

Biqi Pan orcid: 0000-0002-7107-8302.
